# *Cis*-regulatory effects of transposable element insertion/absence polymorphisms in the *Brassica napus* population

**DOI:** 10.1093/hr/uhaf356

**Published:** 2026-04-01

**Authors:** Guoxiang Yuan, Yang Huang, Chunlu Tao, Minxin Li, Qinxia Dai, Yirong Wang, Huaixin Li, Xiangdong Kong, Zongji Zhang, Ri Ming, Li Zhong, Yu Liang, Maoteng Li

**Affiliations:** Key Laboratory of Ecology of Rare and Endangered Species and Environmental Protection, Guangxi Key Laboratory of Landscape Resources Conservation and Sustainable Utilization in Lijiang River Basin, University Engineering Research Center of Bioinformation and Genetic Improvement of Specialty Crops, Guangxi, College of Life Science, Guangxi Normal University, Guilin, China; Xianghu Laboratory, Hangzhou, China; School of Mechanical and Electrical Engineering, Guilin University of Electronic Technology, Guilin, China; Key Laboratory of Ecology of Rare and Endangered Species and Environmental Protection, Guangxi Key Laboratory of Landscape Resources Conservation and Sustainable Utilization in Lijiang River Basin, University Engineering Research Center of Bioinformation and Genetic Improvement of Specialty Crops, Guangxi, College of Life Science, Guangxi Normal University, Guilin, China; Key Laboratory of Ecology of Rare and Endangered Species and Environmental Protection, Guangxi Key Laboratory of Landscape Resources Conservation and Sustainable Utilization in Lijiang River Basin, University Engineering Research Center of Bioinformation and Genetic Improvement of Specialty Crops, Guangxi, College of Life Science, Guangxi Normal University, Guilin, China; Key Laboratory of Ecology of Rare and Endangered Species and Environmental Protection, Guangxi Key Laboratory of Landscape Resources Conservation and Sustainable Utilization in Lijiang River Basin, University Engineering Research Center of Bioinformation and Genetic Improvement of Specialty Crops, Guangxi, College of Life Science, Guangxi Normal University, Guilin, China; Key Laboratory of Ecology of Rare and Endangered Species and Environmental Protection, Guangxi Key Laboratory of Landscape Resources Conservation and Sustainable Utilization in Lijiang River Basin, University Engineering Research Center of Bioinformation and Genetic Improvement of Specialty Crops, Guangxi, College of Life Science, Guangxi Normal University, Guilin, China; Department of Biotechnology, College of Life Science and Technology, Huazhong University of Science and Technology, Wuhan, China; Jiguang Gene Biotechnology Co., Ltd, Nanjing, China; Guilin Branch of Guangxi Academy of Agricultural Science, Guilin Research Centre of Agricultural Sciences, Guilin, China; Guilin Branch of Guangxi Academy of Agricultural Science, Guilin Research Centre of Agricultural Sciences, Guilin, China; Guilin Branch of Guangxi Academy of Agricultural Science, Guilin Research Centre of Agricultural Sciences, Guilin, China; Key Laboratory of Ecology of Rare and Endangered Species and Environmental Protection, Guangxi Key Laboratory of Landscape Resources Conservation and Sustainable Utilization in Lijiang River Basin, University Engineering Research Center of Bioinformation and Genetic Improvement of Specialty Crops, Guangxi, College of Life Science, Guangxi Normal University, Guilin, China; Xianghu Laboratory, Hangzhou, China; Department of Biotechnology, College of Life Science and Technology, Huazhong University of Science and Technology, Wuhan, China

In the genome of *Brassica napus*, transposable elements (TEs) account for 46.07%. Within the plant genome, TEs play crucial roles. For instance, LTR (long terminal repeat) retrotransposons are associated with genes regulating heat stress tolerance and promote alternative splicing of genes. Moreover, TE insertion polymorphisms (TIPs) can produce multiple transcript isoforms, which are associated with variations in multiple agronomic traits and secondary metabolites. Although the genetic diversity of *B. napus* has been extensively studied through whole-genome sequencing [1], research on TEs remains limited.

In the present study, TEs were identified using EDTA [2] and DeepTE [3] separately, revealing that 384 367 LTRs and 20 362 DNA elements constitute the major types of TEs in the *B. napus* genome. Given that TEs are prone to insertion and deletion events, we identified 5816 TEs, encompassing both TIPs and TAPs (TE absence polymorphisms), across the genomes of 503 *B. napus* accessions (Fig. 1A). Notably, relative to SNPs, TIPs and TAPs displayed greater nucleotide diversity across semi-winter, spring, and winter ecotypes of rapeseed, while the inter-ecotype genetic differentiation remained comparable to that estimated from SNPs (Fig. S2A, B). Among the subgenomes, the A genome harbored slightly more TAPs (3931) compared to the C genome (1885). The length distribution of these TEs ranged from 100 to 100 000 kb, primarily concentrated between 100 kb and 1000 kb (Fig. 1B). Recent studies employing expression quantitative trait loci (eQTL) analyses have revealed that TE insertion polymorphisms influence population-specific gene regulatory networks [4]. Therefore, the present study analyzed the *cis*-TE-eQTL in a rapeseed population at 20 and 40 days after flowering (DAF) and identified 3611 and 3495 TE-eQTLs at the two stages, respectively. LTR retrotransposons, particularly those from the *Gypsy* and *Copia* subfamilies, constituted the major contributors to TE-eQTLs in rapeseed, with *Gypsy* elements (20 DAF, 2354; 40 DAF, 2265) contributing more regulatory variants than *Copia* elements (20 DAF, 1239; 40 DAF, 1213) (Fig. 1C).

**Figure 1:**
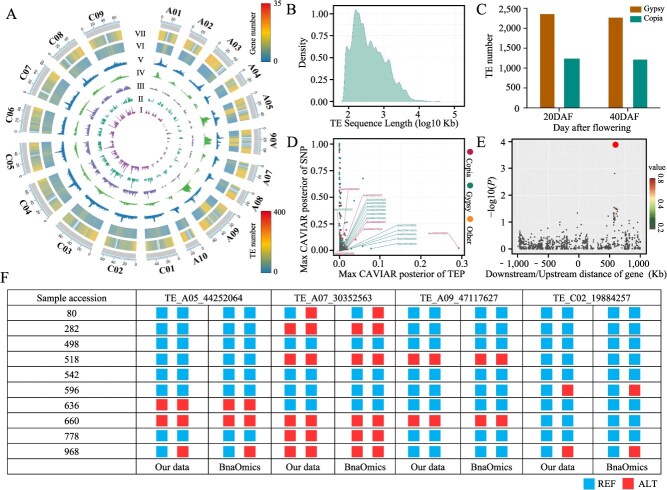
Genome-wide landscape of transposable elements (TEs) and TE-associated regulatory variants in rapeseed. (A) Distribution of transposable elements across the reference genome. Roman numerals denote TE types: I, *POLE*; II, *Helitron*; III, *CACTA*; IV, *Gypsy*; V, *Copia*; VI, number of TEs; VII, number of genes. (B) Length distribution of transposable elements. The x-axis represents TE length, and the y-axis shows the density distribution across different TE size ranges. (C) Numbers of TE-associated eQTLs (TE-eQTLs) identified in rapeseed at 20 and 40 DAF for *Gypsy* and *Copia* elements. The x-axis represents developmental stages (20 and 40 DAF), and the y-axis indicates the number of TE-eQTLs detected at each stage. (D) CAVIAR posterior probabilities for TE-associated eQTLs (TAP-eQTLs) significantly linked to 549 genes (based on TWAS results at 20 DAF). The x-axis indicates the maximum posterior probabilities of TIP/TAP variants, and the y-axis indicates those of SNPs from joint eQTL mapping. Variants located in the lower-right region have higher posterior probabilities for TIP/TAPs than for SNPs. (E) q Values from the joint SNP and TAP-eQTL association analysis for the representative gene *BnaA07G0341600ZS*. Non-significant or non-causal SNPs/TAPs are shown as black points. (F) Comparison between TE genotypes predicted by TEmarker and genotypes with the same structural variation identified by BnaOmics.

In the present study, SNP and TAP genotypes were merged from 503 samples collected at two developmental stages (20 and 40 DAF). In the TWAS analyses [1], the predicted expression levels of lipid-related genes were tested for association with seed oil content at 20 and 40 DAF, revealing 583 and 143 significantly associated genes at each stage, respectively, which suggests stage-specific regulatory mechanisms. To investigate the effects of the TAP and TIP genotypes identified in this study on these candidate genes, we performed *cis*-eQTL mapping by scanning genotype-expression associations within 1 Mb of each gene's transcription start site (TSS). Through this *cis*-eQTL analysis, we identified 3772 gene expression level-associated TE insertion/ absence polymorphisms (TEPs) (Table S1) and 434 909 SNPs at 20 DAF (Table S2), along with 3773 TEPs and 430 612 SNPs at 40 DAF (Table S3). From the 549 lipid-related genes identified in the TWAS analysis of the 20DAF samples, we extracted the maximum posterior probabilities of TEP/SNP_eQTLs (Fig. 1D, Table S4). TEPs, as large-scale structural variants, showed stronger association signals than SNPs at 18 specific loci and were classified as the putative causal variants. Stronger association signals of TEPs likely reflect their capacity as structural variants to reconfigure regulatory architecture, thereby exerting broader effects than SNPs. For example, the causal variant affecting expression of *BnaA07G0341600ZS* (*Cysteine-rich receptor-like protein kinase 10*) was identified as TE_A07_30352563 (LTR/*Gypsy*) (Fig. 1E). In comparison, at 40 DAF, only three TEP eQTLs were identified as putative causal variants relative to SNP-based eQTLs, with one overlapping the TEP eQTL detected at 20 DAF, highlighting the temporal specificity of TAP/TIP-mediated gene expression regulation (Table S5). This study represents a significant discovery in uncovering the functional potential of TEPs in population genomics, thereby necessitating both increased attention to the regulatory roles of TEs in genome evolution and comprehensive mining of population-scale data. The present study provides an exemplary framework for integrating structural variants into causal inference analyses of complex traits. To validate the accuracy of the TEPs identified in this study, we randomly selected 10 transposon deletion variants from the TEP and cross-referenced them with structural variants in the BnaOmics database (https://bnaomics.ocri-genomics.net/), revealing consistent structural variant profiles that further substan tiate the reliability of our TEP results (Fig. 1F).

The population genomics-based identification of TE sequences absent in the reference genome is inherently constrained by limitations such as sequencing depth biases; however, integrating population transcriptome data enables complementary detection of expressed TEs, thereby expanding the characterization of TE dynamics and diversity within the population. Integrated analysis of multi-accession transcriptome datasets encompassing 599 samples [1] enabled systematic pan-transcriptome assembly, obtaining 52 322 novel transcripts absent in the ZS11 genome. Based on the *B. napus* pan-genome [5], we excluded transcripts overlapping presence–absence variations (PAVs) and identified 29 788 novel, non-pangenome transcripts. Functional enrichment analysis (Fisher's exact test, FDR < 0.05) indicated that these transcripts are highly enriched in growth- and development-related pathways (Fig. S1A). For example, nine non-pangenome transcripts are involved in the mTOR signaling pathway, which is related to cell growth and division. These include transcripts encoding TELO2, NPRL2, WDR24/SEA2, and LAMTOR2/4, all of which are established regulators of the TORC1 signaling pathway. GO enrichment analysis revealed that non-pangenome transcripts were enriched in metabolite synthesis-related GO terms (Fig. S1B), such as cholesterol metabolic process (133 non-pangenome transcripts). Sequence alignment of non-pangenome transcripts against our genome-annotated TE reference library identified 16 257 TE-derived transcripts. Non-pangenome transcripts were aligned to the curated TE reference library using GMAP and only matches with ≥80% sequence identity, ≥50 bp alignment length, and ≤5 mismatches were considered TE-derived (see Supplementary Method S2 for details). While current methodologies preclude chromosomal anchoring of these sequences, our transcriptome-wide analysis expands the characterization of TIPs, providing comprehensive and refined population-level abundance estimates of transcriptionally active TIPs.

Overall, this study investigates the variation characteristics of TEs in the *B. napus* at both genomic and transcriptomic levels, offering new insights into the role of TE variations in the *B. napus*. This research lays a foundation for further understanding and utilizing the features of TEs to facilitate crop improvement.

## Supplementary Material

Web_Material_uhaf356

## Data Availability

Data sequences can be downloaded from figshare (https://doi.org/10.6084/m9.figshare.30579425). The code used in this study is available at the following website: (https://doi.org/10.6084/m9.figshare.30608039). Supplementary data can be downloaded from figshare (https://doi.org/10.6084/m9.figshare.30931352).
