# Supplementary material for: Cis-regulatory effects of transposable element insertion/absence polymorphisms in the Brassica napus population
Source: Hortic Res. 2026 Apr 1;13(4):uhaf356. doi: 10.1093/hr/uhaf356 (PMC13365621; doi:10.1093/hr/uhaf356)
Supplement: Web_Material_uhaf356 [file web_material_uhaf356.zip › Supplementary data/Supplementary data.R1.docx]

**Supplementary data**

Table S1 Summary of TEP eQTLs.

Table S2 20DAF significant TE_eQTLs

Table S3 40DAF significant TE_eQTLs

Table S4 The casual TEP of lipid-related genes in 20DAF

Table S5 The casual TEP of lipid-related genes in 40DAF

Figure S1: Pan-transcriptome construction and analysis. KEGG (A) and GO (B) enrichment results of non-pangenome transcripts.

Figure S2: Comparison of pi and FST of SNPs (A) and TEs (B) among different rapeseed populations

**Supplemental Method S1. Transposable element insertion/absence polymorphism identification**

This study utilized 505 genome resequencing datasets from the PRJCA002835 project (NCBI SRA database; Tang et al., 2021). The initial data processing involved two steps. First, raw SRA format data were converted to fastq format using the SRA Toolkit's fastq-dump. Second, quality control was performed on the fastq data using fastp (Chen et al., 2018).

The workflow for transposable element (TE) analysis comprised two main phases.

Phase 1: Construction of a high-confidence TE library. We used the ZS11 genome as the reference (Song et al., 2020). First, a preliminary TE annotation was generated using the EDTA pipeline (Ou et al., 2019). Next, sequences classified as "LTR/unknown" by EDTA were extracted and further analyzed with the deep learning-based tool DeepTE (Yan et al., 2020). Finally, the DeepTE classification results were integrated into the TE library, and unclassifiable sequences were removed to produce a refined, high-confidence library.

Phase 2: Detection and genotyping of TE insertion/absence polymorphisms. To begin, we identified TE insertions and deletions in each sample using the TEMP2 pipeline (Yu et al., 2021). Subsequently, TE genotyping was performed with TEmarker (Yan et al., 2022). Specifically, for TEmarker analysis, the insertion and deletion calls from TEMP2 were merged and converted into BED format to create the required input file. This two-phase approach allowed for the comprehensive identification and genotyping of TIP/TAP across all samples.

**Supplemental Method S2. TAP-eQTL analysis and fine mapping**

Variant filtering and TE-eQTL mapping were performed as follows. VCF files were first filtered using VCFtools v0.1.17 to retain variants with a minor allele frequency (MAF) greater than 0.01. FastQTL v2.184 was then employed to test for associations between genetic variants and transcript expression within a 1 Mb window centered on the transcription start site (TSS). The analysis incorporated genotype-derived principal components as covariates, and expression values were subjected to rank-based inverse normal transformation to approximate normality. Nominal P-values were obtained and adjusted for multiple testing using the Benjamini–Hochberg false discovery rate (FDR). For fine-mapping, z-scores were derived from the estimated effect sizes (beta) and P-values, and these were supplied to CAVIAR (Hormozdiari et al., 2014), together with linkage disequilibrium (LD) information, to infer the posterior causal probability of each variant.

**Supplemental Method S3. Pan-transcriptome assembly**

In this study, 599 RNA-seq datasets (PRJCA002835) were aligned to the ZS11 reference genome using STAR v2.7.1 (Dobin et al., 2013), followed by the extraction of unmapped reads with samtools v1.9 (Li et al., 2009). Trinity v2.11 was employed for the de novo assembly of unmapped reads (Grabherr et al., 2011). Redundancies were removed using cd-hit v4.8.1 (default parameters) (Fu et al., 2012), and sequences not belonging to green plants were excluded based on species classification information after alignment with the NT database using blastn (https://blast.ncbi.nlm.nih.gov/blast/Blast.cgi). Transcripts shorter than 200 bp were subsequently discarded. These transcripts were mapped to the reference genome and new sequences of the pan-genome using GMAP (Wu and Watanabe, 2005), and transcripts with a coverage of <85% were filtered out. For the remaining transcripts, blastn (with an e-value threshold of 1e-5) was used to align them to the non-reference coding sequence (CDS) of pan-genome, excluding transcripts that aligned to non-reference CDS of pan-genome (Song et al., 2021). The remaining transcripts were considered as final novel transcripts.

TransDecoder.LongOrfs (https://github.com/TransDecoder/TransDecoder) was utilized to predict open reading frames (ORF) in the novel transcripts. Following the identification of CDS, the predicted CDS were annotated functionally by alignment with the SwissProt database using BLASTX. Protein sequences were annotated with pfam domains using hmmsearch (http://hmmer.org/). Functional enrichment analysis of the novel transcripts was conducted using the clusterProfiler R package (Wu et al., 2021), and the results of GO enrichment were visualized with CirGO (Kuznetsova et al., 2019).

Reference:

**Chen S, Zhou Y, Chen Y, Gu J** (2018) Fastp: An ultra-fast all-in-one FASTQ preprocessor. Bioinformatics **34**: i884–i890

**Dobin A, Davis CA, Schlesinger F, Drenkow J, Zaleski C, Jha S, Batut P, Chaisson M, Gingeras TR** (2013) STAR: ultrafast universal RNA-seq aligner. Bioinformatics **29**: 15–21

**Fu L, Niu B, Zhu Z, Wu S, Li W** (2012) CD-HIT: accelerated for clustering the next-generation sequencing data. Bioinformatics **28**: 3150–3152

**Grabherr MG, Haas BJ, Yassour M, Levin JZ, Thompson DA, Amit I, Adiconis X, Fan L, Raychowdhury R, Zeng Q, et al** (2011) Full-length transcriptome assembly from RNA-Seq data without a reference genome. Nat Biotechnol **29**: 644–652

**Hormozdiari F, Kostem E, kang EY, Pasaniuc B, Eskin E** (2014) Identifying Causal Variants at Loci with Multiple Signals of Association. Proc. 5th ACM Conf. Bioinformatics, Comput. Biol. Heal. Informatics. Association for Computing Machinery, New York, NY, USA, pp 610–611

**Kuznetsova I, Lugmayr A, Siira SJ, Rackham O, Filipovska A** (2019) CirGO: an alternative circular way of visualising gene ontology terms. BMC Bioinformatics **20**: 84

**Li H, Handsaker B, Wysoker A, Fennell T, Ruan J, Homer N, Marth G, Abecasis G, Durbin R** (2009) The Sequence Alignment/Map format and SAMtools. Bioinformatics **25**: 2078–2079

**Ongen H, Buil A, Brown AA, Dermitzakis ET, Delaneau O** (2016) Fast and efficient QTL mapper for thousands of molecular phenotypes. Bioinformatics **32**: 1479–1485

**Ou S, Su W, Liao Y, Chougule K, Agda JRA, Hellinga AJ, Lugo CSB, Elliott TA, Ware D, Peterson T, et al** (2019) Benchmarking transposable element annotation methods for creation of a streamlined, comprehensive pipeline. Genome Biol **20**: 275

**Song JM, Guan Z, Hu J, Guo C, Yang Z, Wang S, Liu D, Wang B, Lu S, Zhou R, et al** (2020) Eight high-quality genomes reveal pan-genome architecture and ecotype differentiation of Brassica napus. Nat Plants **6**: 34–45

**Song JM, Liu DX, Xie WZ, Yang Z, Guo L, Liu K, Yang QY, Chen LL** (2021) BnPIR: Brassica napus pan-genome information resource for 1689 accessions. Plant Biotechnol J **19**: 412–414

**Tang S, Zhao H, Lu S, Yu L, Zhang G, Zhang Y, Yang Q-Y, Zhou Y, Wang X, Ma W, et al** (2021) Genome- and transcriptome-wide association studies provide insights into the genetic basis of natural variation of seed oil content in Brassica napus. Mol Plant **14**: 470–487

**Wu T, Hu E, Xu S, Chen M, Guo P, Dai Z, Feng T, Zhou L, Tang W, Zhan L, et al** (2021) clusterProfiler 4.0: A universal enrichment tool for interpreting omics data. Innovation **2**: 100141

**Wu TD, Watanabe CK** (2005) GMAP: a genomic mapping and alignment program for mRNA and EST sequences. Bioinformatics **21**: 1859–1875

**Yan H, Bombarely A, Li S** (2020) DeepTE: a computational method for de novo classification of transposons with convolutional neural network. Bioinformatics **36**: 4269–4275

**Yan H, Haak DC, Li S, Huang L, Bombarely A** (2022) Exploring transposable element-based markers to identify allelic variations underlying agronomic traits in rice. Plant Commun **3**: 100270

**Yu T, Huang X, Dou S, Tang X, Luo S, Theurkauf WE, Lu J, Weng Z** (2021) A benchmark and an algorithm for detecting germline transposon insertions and measuring de novo transposon insertion frequencies. Nucleic Acids Res **49**: e44–e44
