# Supplementary figures and images for: Cis-regulatory effects of transposable element insertion/absence polymorphisms in the Brassica napus population
Source: Hortic Res. 2026 Apr 1;13(4):uhaf356. doi: 10.1093/hr/uhaf356 (PMC13365621; doi:10.1093/hr/uhaf356)

A

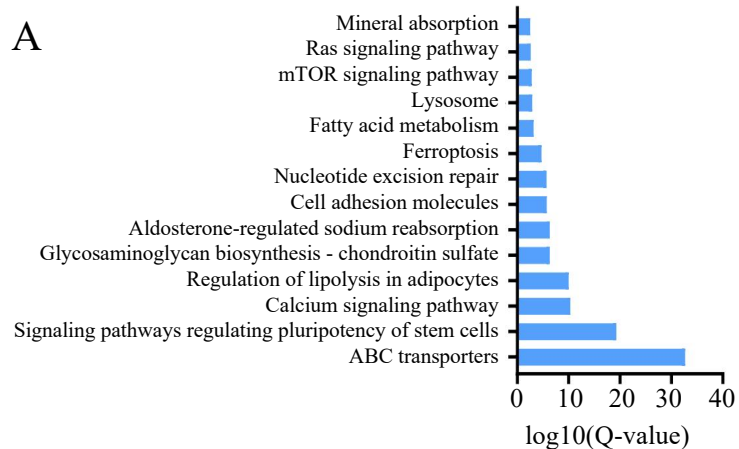

B

Name and Proportion of the Biological Process (Inner Ring)

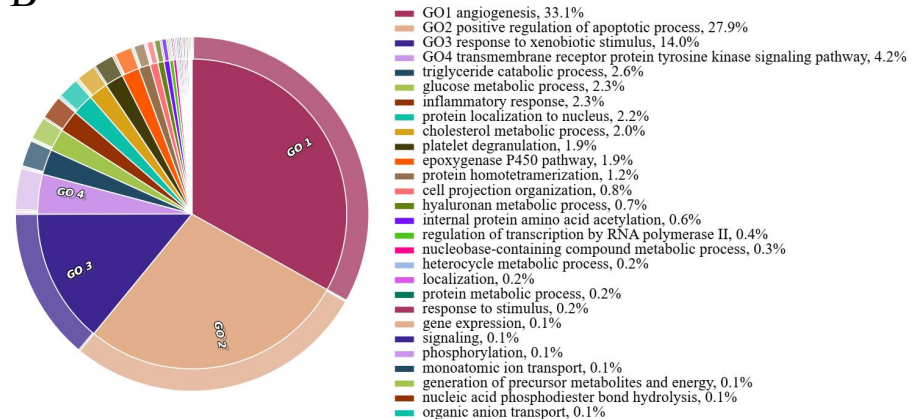

Supplement: Web_Material_uhaf356 [file web_material_uhaf356.zip › Supplementary data/Supplementary Figure S1.pdf]

**A**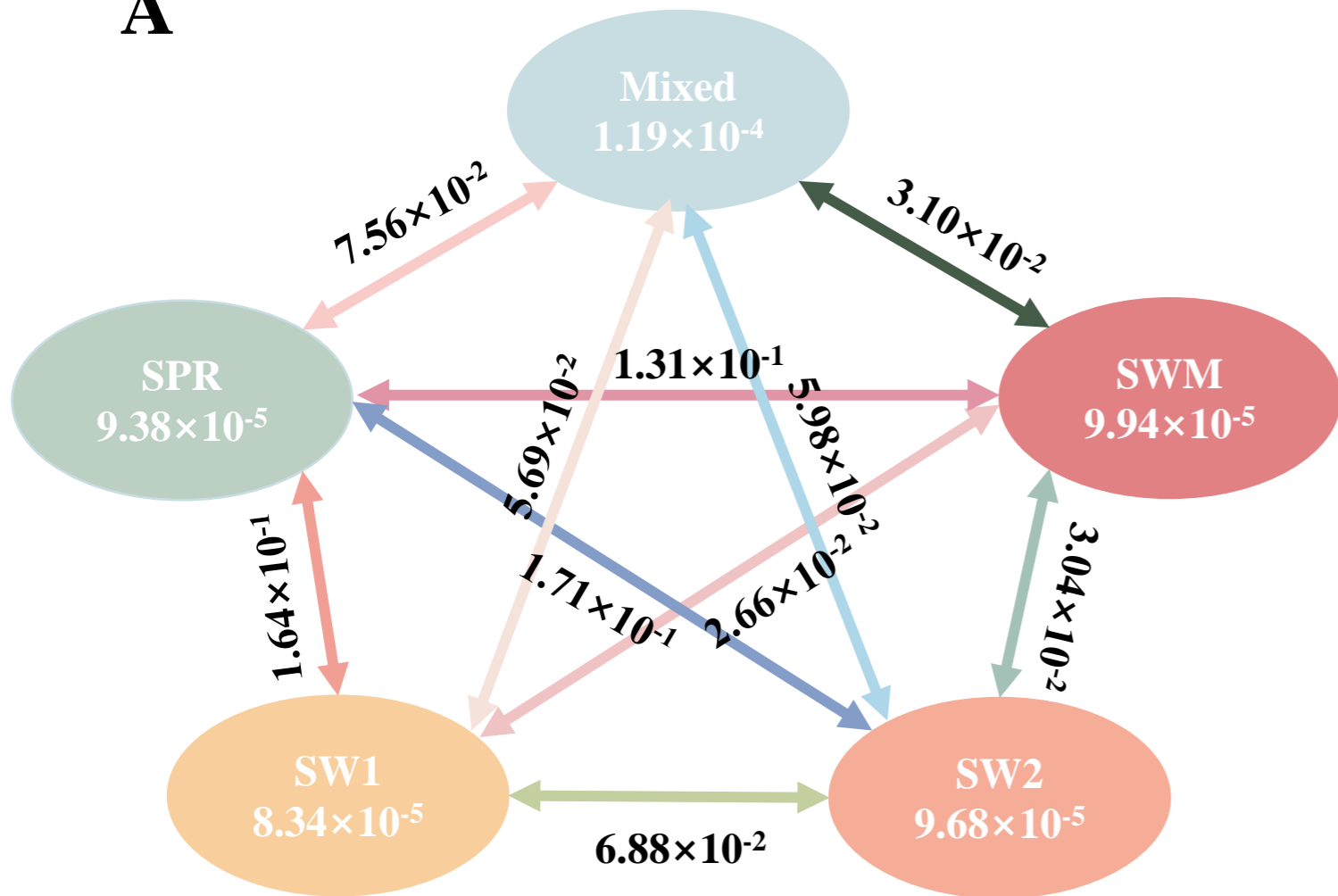**B**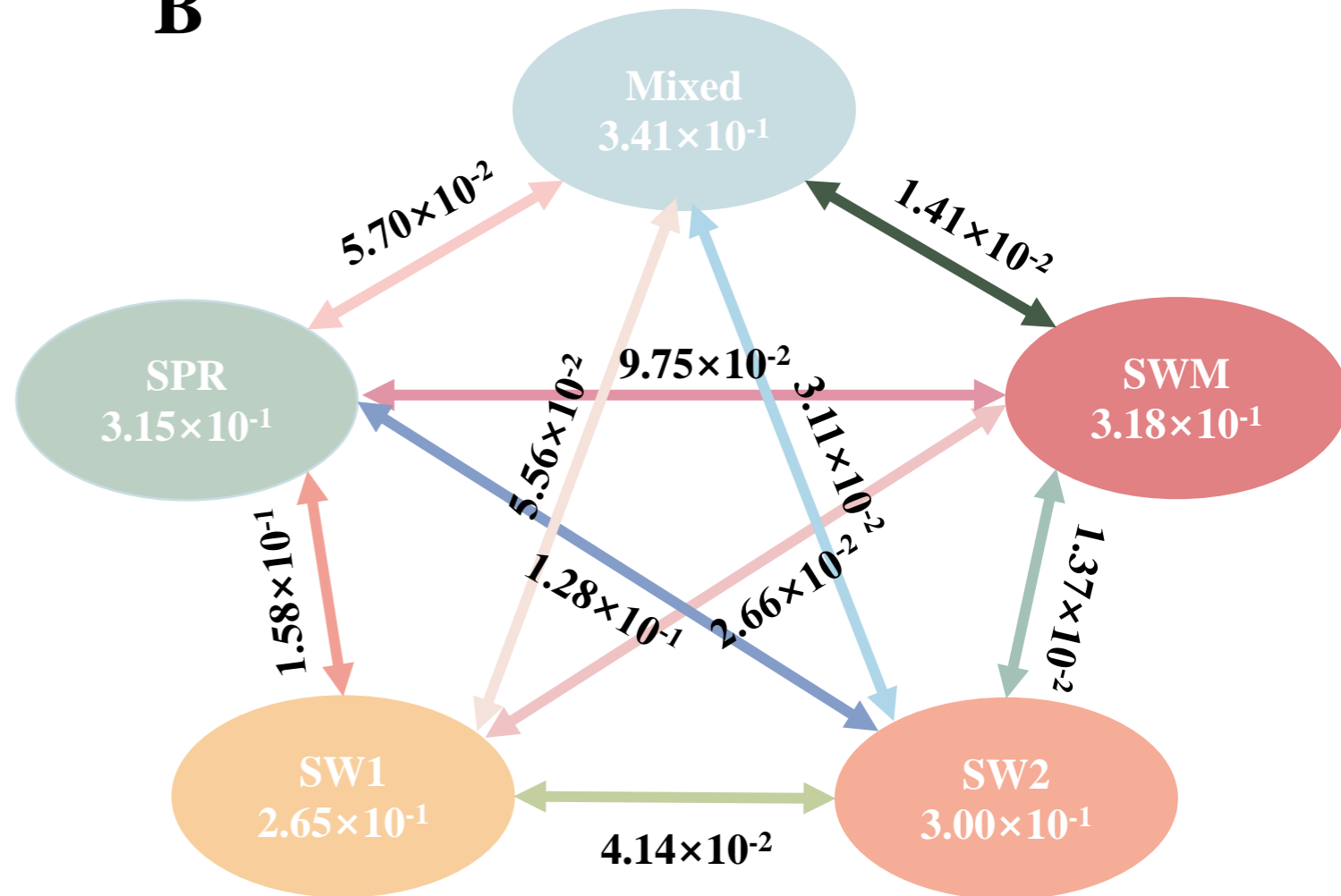

Supplement: Web_Material_uhaf356 [file web_material_uhaf356.zip › Supplementary data/Supplementary Figure S2.pdf]
